# Robustness of Breast Margins with Volumetric Modulated Arc Therapy without a Six-Degrees-of-Freedom Couch: A Dosimetric Evaluation

**DOI:** 10.3390/jcm12030862

**Published:** 2023-01-21

**Authors:** Jessica Prunaretty, Nicolas Mir, Anaïs Tilhac, Maureen Gouillou, David Azria, Pascal Fenoglietto, Céline Bourgier

**Affiliations:** Department of Radiation Oncology, Montpellier Cancer Institute, 34298 Montpellier, France

**Keywords:** VMAT, breast cancer, planning margins

## Abstract

In our hospital, a TrueBeam linear accelerator and the PerfectPitch 6-degrees-of-freedom (6-DOF) couch (Varian), with 7 mm margins, are used for volumetric modulated arc therapy (VMAT) of breast cancer (BC). This study tested whether a 3-degrees-of-freedom (3-DOF) couch, i.e., without rotation compensation (such as the Halcyon couch), affected dose metrics. A total of 446 daily extended cone beam computed tomography (CBCT) data of 20 patients who received VMAT for BC were used to recalculate the treatment plans with the session registration (6-DOF) and a simulated matching with 3-DOF. The initial plan provided significantly better coverage for internal mammary chain and clavicular lymph node clinical target volumes (CTVs) than the 6-DOF and 3-DOF CBCT plans. The volumes receiving 110% of the prescribed dose (V110%) were increased for all CTVs with the 6-DOF and 3-DOF CBCT plans, but the difference was significant only for the breast/chest wall CTV (*p* < 0.05; paired samples *t*-test). Protection of the heart and lungs was comparable among plans. The dose volume histograms based on the 6-DOF and 3-DOF data were similar for CTVs and organs at risk. Therefore, with a 7 mm margin, VMAT and a 3-DOF couch can be used for BC treatment without any compromise in delivery accuracy.

## 1. Introduction

Currently, breast-conserving surgery followed by radiation therapy is the standard treatment for early breast cancer. Although the American Society for Radiation Oncology (ASTRO) does not recommend intensity-modulated radiation therapy (IMRT) for the routine delivery of whole-breast irradiation following breast-conserving surgery, its use is increasing worldwide [1,2].

Due to IMRT higher plan conformity compared with three-dimensional conformal radiotherapy [3] and the inter-fraction variability in breast volumes [4,5], the choice of appropriate margins between clinical target volume (CTV) and planning target volume (PTV) is a real concern. The Radiation Therapy Oncology Group (RTOG) recommends a uniform 7 mm margin from CTV to PTV [6]. However, several studies have shown that margins are influenced by many different factors, such as patient characteristics and choice of image-guided radiation therapy technique [7,8]. Rossi et al. [9] evaluated the dosimetric effects of breast deformation using 2D and 3D matching methods and recommended daily automatic cone beam computed tomography (CBCT) matching of the volumetric modulated arc therapy (VMAT) treatment plans. The patient set-up accuracy is also improved by using six-degrees-of-freedom (6-DOF) couches compared with 4-DOF couches (without the pitch and roll rotational movements). Indeed, it has been reported [10,11,12] that the absence of rotational compensation leads to a loss of PTV dose coverage. Mancosu et al. [12] studied pitch and roll compensations offered by the 6-DOF couch at different treatment sites by analyzing 2945 fractions for 376 consecutive patients. They concluded that these compensations improve the patient set-up at all treatment sites, particularly the brain.

At the Cancer Institute of Montpellier, France, VMAT in patients with breast cancer is performed using a TrueBeam linear accelerator with the PerfectPitch^TM^ 6-DOF couch (Varian, Medical Systems, Palo Alto, CA, USA) and following RTOG margin recommendations. After the recent acquisition of a Halcyon machine equipped with a 3-DOF couch (without rotational correction), we evaluated the dosimetric feasibility of using VMAT for breast cancer treatment with the recommended margins but without rotation compensation.

## 2. Materials and Methods

### 2.1. Patient Selection

Twenty patients treated for invasive breast carcinoma between November 2021 and December 2022 were prospectively enrolled and divided into four equal treatment groups: right breast, left breast, right chest wall, and left chest wall (walls included regardless of the breast reconstruction status). Patients were included regardless of age, histology, tumor grade, surgical treatment (lumpectomy or mastectomy), or neoadjuvant chemotherapy. Patient characteristics are described in Table 1.

### 2.2. Treatment Planning

Patients underwent Computed Tomography (CT) imaging (GE Optima CT580, General Electric Healthcare, Waukesha, WI, USA), at a 2.5 mm-slice thickness, in supine position, free breathing, and both arms over the head with personalized foam cushions.

The ESTRO consensus guidelines [13,14] were used to delineate target volumes, breast/wall, and axillary (Berg I); subclavicular (Berg II, III) and supraclavicular (Berg IV) lymph nodes (Nodes hereafter); and internal mammary chain (IMC). The PTV was defined as a 3D expansion of the CTV with a margin of 7 mm [6]. All PTVs and CTVs were limited to 5 mm under the skin. Organs at risk were delineated following French RecoRad 2022 [15] recommendations using TheraPanacea software [16].

Patients were treated with a simultaneous integrated boost and a dose prescription of 63.22 Gy in 29 fractions to the tumor bed, 52.2 Gy to the breast and to the IMC, and 49.3 Gy to the clavicular lymph node region. VMAT optimization and calculation were performed with the treatment planning system Eclipse (Varian, Medical Systems, Palo Alto, CA, USA), Photon Optimizer (PO, v15.6, Varian, Medical Systems, Palo Alto, CA, USA), and the analytical anisotropic algorithm (AAA, v15.6), respectively. Treatment was delivered by at least two partial arcs (6 MV photons, 400 UM/min dose rate) and normalized as such that 99% of the breast/chest wall CTVs received 49.6 Gy (i.e., 95% of the prescribed dose to the breast/chest wall).

**Figure 1:**
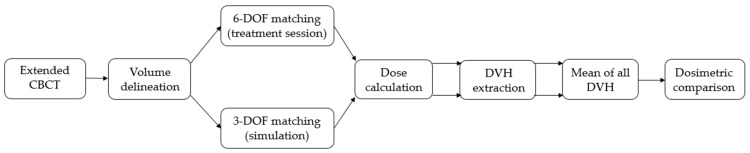
Workflow of the dosimetric comparison between 6-DOF and 3-DOF CBCT plans.

All patients were irradiated using a TrueBeam linear accelerator associated with the PerfectPitchTM 6-DOF couch. Daily extended CBCT was performed for the patient set-up using the TrueBeam on-board imaging system (Varian Medical System) to evaluate the dose distributions to all CTVs, such as for the heart and lungs. The dosimetric effects of no patient rotation were evaluated by simulating a 3-DOF matching. The workflow is described in Figure 1. First, the physician delineated all CTVs, heart and lungs, on each CBCT. Two CBCT plans were created: 6-DOF registration (performed by the therapists during the treatment session) and 3-DOF registration (performed by the physician). The matching procedure was similar for both registrations: first, an automatic bone match; then, a manual adjustment (if necessary) to include the CTVs in the PTV structures. Dose was calculated using the same original control points in the Eclipse treatment planning system. Note that the Eclipse treatment planning system (TPS) does not perform calculations on rotated CT images. When rotations were involved, the field isocenter was positioned by the TPS with the 6-DOF registration using the Eulersche Transformation matrix. The dose volume histograms (DVHs) of each session were extracted and averaged.

### 2.3. Plan Analysis

First, information on pitch, roll, and yaw rotations applied during the treatment sessions (6-DOF registration) was collected. The mean values and standard deviations were calculated and the absolute values were recorded.

Then, the breast and chest wall volumes of each CBCT were analyzed to evaluate deformation during treatment.

Dose metrics were compared between the 6-DOF and 3-DOF CBCT plans and the initial plan. Mean and standard deviations were extracted from the DVH parameters. For the CTVs (breast/chest wall, IMC, and other lymph nodes), the volumes receiving 95% and 110% of the prescribed dose (V95% and V110%) were evaluated. For the ipsilateral lung, doses received at 10%, 20%, and 80% of the volume (D10%, D20%, D80%) were calculated. The mean doses to the heart and contralateral lung were also recorded. Matching methods were compared with the paired samples *t*-test, and a *p*-value of < 0.05 was considered significant. Statistical analyses were conducted using Microsoft Excel.

Lastly, the rate of treatment sessions with unacceptable CTV coverage was evaluated. The criterion for non-acceptability of a treatment plan was <95% of the whole CTV (breast/chest wall, IMC and nodes) covered by 95% of the prescribed dose. If this criterion was not satisfied for one of the irradiated areas, the treatment plan was considered not clinically acceptable.

## 3. Results

**Figure 2:**
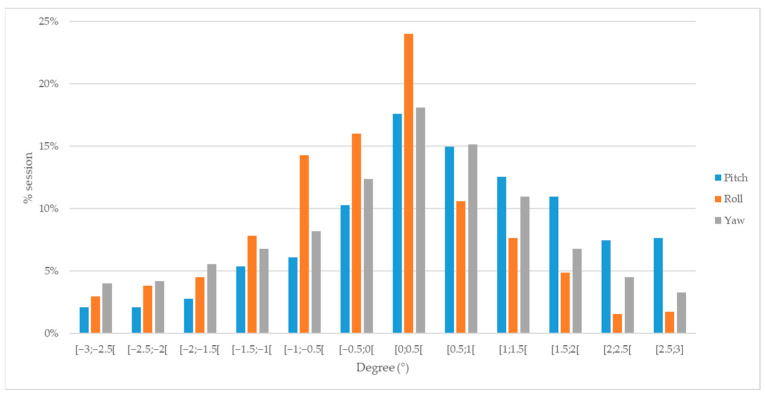
Histogram showing the distribution of pitch (blue), roll (orange), and yaw (grey) adjustments of each treatment session.

This study included 446 treatment sessions for 20 patients. In total, 134 CBCT data were excluded because extended images were missing. One patient needed their treatment plan altering during treatment and 1.7% of sessions required a physical repositioning of the patient. The mean rotational set-up adjustments were 0.56°± 1.24°, −0.13°± 1.16°, and 0.14° ± 1.35° for pitch, roll, and yaw, respectively, with normally distributed data (Figure 2). The mean absolute values were 1.09° ± 0.82°, 0.88° ± 0.77°, and 1.08° ± 0.83° for pitch, roll, and yaw rotations, respectively.

**Figure 3:**
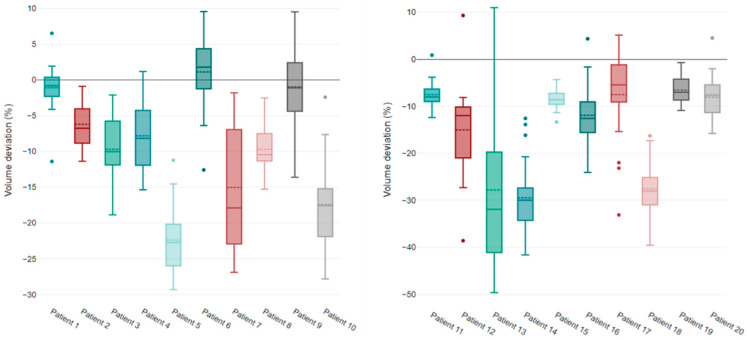
Box plot showing the variation in breast (**left**) and chest wall (**right**) CTV values in each patient (*n* = 20) during treatment.

**Figure 4:**
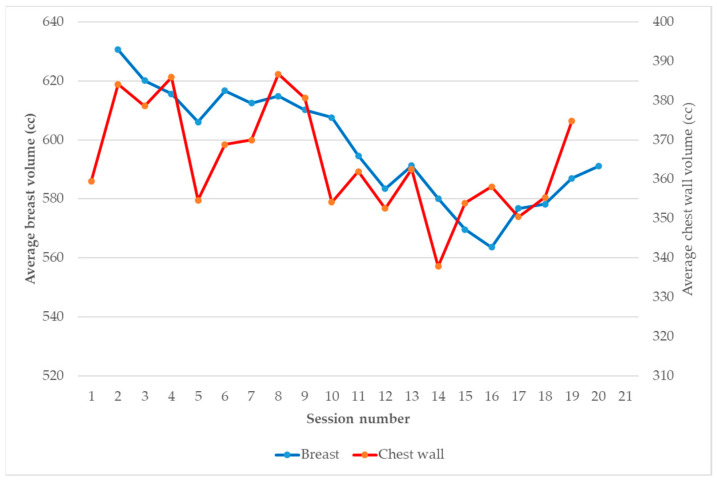
Evolution of the breast (blue line) and chest wall (red line) CTV volume during the sessions.

Considering the breast and chest wall inter-fraction variation, the CTVs were delineated by the physician for each CBCT and analyzed individually (Figure 3). Volume variations were more important for the chest wall volume than the breast volume: mean absolute deviations relative to the median value of 8.57% ± 5% and 4.3% ± 2.03%, respectively. However, these variations did not show a specific trend (up or down), as shown in Figure 4.

**Figure 5:**
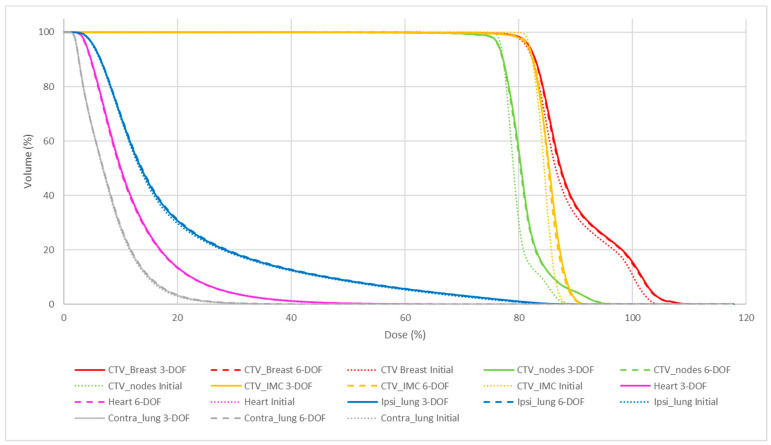
Dose volume histograms for the initial plan (dotted lines), 6-DOF (dashed lines), and 3-DOF (solid lines) CBCT plans for breast irradiation. N.B. The breast CTV includes the tumor bed (100% of the prescribed dose), which was not evaluated in this study.

**Figure 6:**
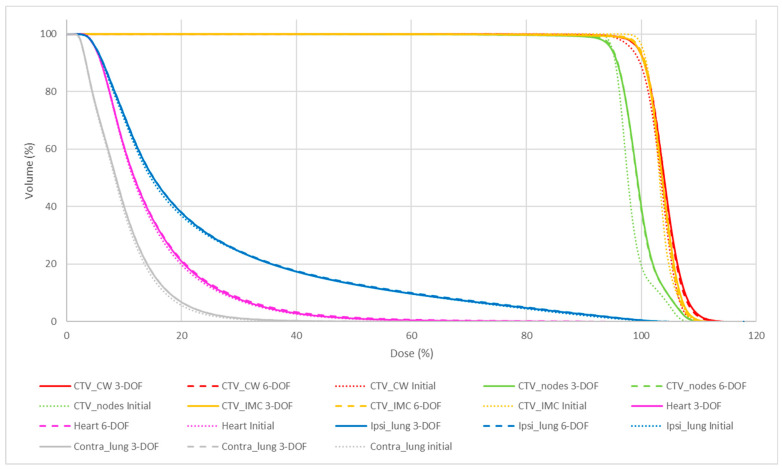
Dose volume histograms for the initial plan (dotted lines), and 6-DOF (dashed lines) and 3-DOF (solid lines) CBCT plans for chest wall irradiation.

Table 2 and Table 3, and Figure 5 and Figure 6 summarize the quality of the initial plans and the CBCT plans with 6-DOF and 3-DOF matching for breast and chest wall localization. A comparison of the DVH parameters (*p*-values) of the different plans and matching methods is presented in Table 2. The initial plan provided significantly better coverage for CTV_nodes and CTV_IMC compared with the 6-DOF and 3-DOF CBCT plans. The V110% values of all CTVs were increased in the 6-DOF and 3-DOF CBCT plans compared with the initial plan; however, only the V110% of the breast/chest wall CTV was significantly different (*p* < 0.05) among plans with larger standard deviations. The D80% values of the ipsilateral lung were statistically different between the initial plans and both matchings. Nevertheless, the absolute deviation was lower than 0.2 Gy. Finally, the protection of the heart and lungs was comparable.

However, the dosimetric differences were not dependent on the matching method. Indeed, the DVHs obtained with the 6-DOF and 3-DOF registration methods were similar for the CTVs and organs at risk.

No difference was observed between breast and chest wall irradiation.

The unacceptable treatment session rate was not significantly different between the 3-DOF and 6-DOF CBCT plans (*p* = 0.513). CTV coverage was acceptable in 89.69% of treatment sessions with 6-DOF matching and 88.12% of treatment sessions with 3-DOF matching.

## 4. Discussion

To date, VMAT of breast cancer has been performed in our department with a TrueBeam linear accelerator and the PerfectPitch 6-DOF couch (Varian, Medical Systems, Palo Alto, CA, USA) using 7 mm margins. With the recent acquisition of a Halcyon machine equipped with a 3-DOF couch, the feasibility of treating VMAT breast cancer without rotation compensation was questioned. The aim of this study was to evaluate the robustness of the 7 mm breast cancer VMAT margins when using 3-DOF matching.

The analysis of the mean pitch, roll, and yaw adjustments showed no systematic errors. Jiang et al. [17] compared the set-up errors between an optical imaging system and CBCT for breast radiotherapy with equivalent patient positioning (two arms above the head combined with a vacuum). Their results were consistent with ours: 0.83 ± 0.7°, 1.12 ± 0.79°, and 1.07 ± 0.81° in pitch, roll, and yaw directions, respectively (1.15° ± 0.85°, 0.88° ± 0.76°, and 1.06° ± 0.82°, in our study).

Under the considered experiments, dosimetric differences were highlighted for the target volumes between the initial plans and the CBCT plans based on the 6-DOF or 3-DOF matching, but not between the 6-DOF and 3-DOF CBCT plans. The dosimetric influence of patient rotational set-up errors was statistically insignificant when corrected by couch adjustments. The target coverage and the dose to the organs at risk were comparable with both matching methods, showing the robustness of the 7 mm margins. These observations seem to suggest that the anatomical deformations rather than the positioning method have a more important impact. It is known that breast deformations are common during radiotherapy treatment [18,19]. Several studies have recommended the use of the skin flash planning technique to factor in anatomical variations to ensure VMAT dosimetry robustness [7,8,9,10,11,12,13,14,15,16,17,18,19,20]. In our department, we chose to perform a daily CBCT and to adapt the registration method to the anatomical deformation. Matching to the skin or pulmonary wall is undertaken based on swelling or shrinkage breast cases, respectively. When tissue deformations exceed 10 mm, replanning is needed.

The main limitation of this study was the manual delineation of the target volumes. These biases were studied by H. Struikmans et al. in 18 patients who underwent conservative breast surgery [21]. The CTVs were delineated by five different observers. For one observer, the breast CTV was higher than the mean value in 17/18 patients, and for another observer, in 10/18 patients. This demonstrates the importance in determining beforehand the inter-observer variability to recognize the impact of manual contouring on the structure volumes. Despite delineation guidelines [13,14], Song et al. demonstrated residual contouring variations in the quality assurance results of the pretrial benchmark case for the POTENTIAL trial [22]. We limited this variability definition with only two observers to delineate. Moreover, a warning was sent when the ratio with the initial contour exceeded 50%. The tumor bed CTV was not studied for similar reasons. Although a standardized breast tumor bed surgical clipping protocol was used to enable accurate localization of the CTV, shrinkage of the boost volume is common during radiation therapy [23,24]. In addition, accurately localizing the tumor bed on CBCT images can be challenging.

Our study focused on the dosimetric feasibility of using 3-DOF matching in VMAT for breast cancer with 7 mm margins, based on a recalculation of CBCT images. This method can be used to evaluate the dose distribution for breast cancer and has already been employed to clinically select patients with breast cancer requiring adaptive treatment [25].

## 5. Conclusions

With 7 mm margins, it is possible to use a 3-DOF couch for patients with breast cancer receiving VMAT without any compromise in delivery accuracy compared with a 6-DOF couch. The dosimetric influence of the patient rotational set-up errors can be corrected by couch translation. Future studies should evaluate the possible margin reduction with a potential offline/online plan adaption strategy.

## Figures and Tables

**Table 1:** Patient characteristics, including treatment side, type of surgery, and volume of breast/chest wall CTV.

Patient	Laterality	Type	CTV Breast/Chest Wall Volume (cc)
1	Right	Conserving surgery	802.5
2	Right	Conserving surgery	489.7
3	Right	Conserving surgery	904.6
4	Right	Conserving surgery	501.2
5	Right	Conserving surgery	381.5
6	Left	Conserving surgery	575.0
7	Left	Conserving surgery	754.2
8	Left	Conserving surgery	692.5
9	Left	Conserving surgery	870.3
10	Left	Conserving surgery	475.4
11	Right	Mastectomy	415.9
12	Right	Mastectomy	274.0
13	Right	Mastectomy	527.8
14	Right	Mastectomy	250.3
15	Right	Mastectomy	516.1
16	Left	Mastectomy	384.5
17	Left	Mastectomy	237.6
18	Left	Mastectomy	368.5
19	Left	Mastectomy	621.1
20	Left	Mastectomy	623.6

**Table 2:** Comparison of dose metrics for the initial and 3-DOF and 6-DOF CBCT treatment plans.

			Breast	Chest Wall
CTV Breast	V_95%_ (%)	Initial	99.07 ± 0.15	99.06 ± 0.17
		6-DOF	99.26 ± 0.75	99.17 ± 0.61
		3-DOF	99.17 ± 1.00	99.28 ± 0.53
	V_110%_ (%)	Initial	27.53 ± 13.10	0.10 ± 0.13
		6-DOF	30.07 ± 13.85	1.71 ± 4.37
		3-DOF	30.49 ± 14.26	2.15 ± 5.47
CTV_IMC	V_95%_ (%)	Initial	100 ± 0.00	100 ± 0.00
		6-DOF	99.27 ± 1.08	99.35 ± 0.65
		3-DOF	99.06 ± 1.39	99.11 ± 1.03
	V_110%_ (%)	Initial	0 ± 0.00	0.11 ± 0.27
		6-DOF	0.56 ± 1.12	0.28 ± 0.40
		3-DOF	0.39 ± 0.59	0.59 ± 1.48
CTV_Nodes	V_95%_ (%)	Initial	100 ± 0.00	100 ± 0.00
		6-DOF	99.37 ± 0.61	99.43 ± 0.56
		3-DOF	99.54 ± 0.55	99.21 ± 0.88
	V_110%_ (%)	Initial	6 ± 13.50	2.79 ± 6.67
		6-DOF	9.40 ± 24.23	6.16 ± 8.98
		3-DOF	9.24 ± 23.54	5.79 ± 8.34
Heart	D_mean_ (Gy)	Initial	7.58 ± 1.05	7.65 ± 1.22
		6-DOF	7.51 ± 1.15	7.92 ± 1.48
		3-DOF	7.58 ± 1.09	7.84 ± 1.20
Ipsi_Lung	D_mean_ (Gy)	Initial	12.47 ± 1.31	12.54 ± 1.06
		6-DOF	12.76 ± 1.23	12.79 ± 1.27
		3-DOF	12.71 ± 1.48	12.83 ± 1.17
	D_10%_ (Gy)	Initial	28.75 ± 3.03	31.18 ± 2.23
		6-DOF	29.50 ± 2.72	31.59 ± 2.41
		3-DOF	29.31 ± 3.80	31.79 ± 2.93
	D_20%_ (Gy)	Initial	17.94 ± 2.37	18.77 ± 1.93
		6-DOF	18.16 ± 1.92	18.82 ± 2.17
		3-DOF	18.07 ± 2.55	19.03 ± 2.32
	D_80%_ (Gy)	Initial	5.17 ± 1.03	4.43 ± 0.95
		6-DOF	5.23 ± 1.03	4.55 ± 1.00
		3-DOF	5.21 ± 1.05	4.55 ± 0.98
Contra_lung	D_mean_ (Gy)	Initial	5.00 ± 0.57	5.04 ± 0.37
		6-DOF	5.09 ± 0.57	5.23 ± 0.43
		3-DOF	5.06 ± 0.61	5.12 ± 0.41

**Table 3:** *p*-values for differences in dose metrics between the indicated plans. Pairwise comparisons performed between the initial plan and those obtained with the 3-DOF and 6-DOF, between the plans obtained with the two matching techniques. Values are bolded where *p* < 0.05.

		Initial vs. 6-DOF	Initial vs. 3-DOF	6-DOF vs. 3-DOF
CTV Breast/Chest wall	V_95%_	0.326	0.381	0.951
	V_110%_	**0.014**	**0.015**	0.071
CTV_Nodes	V_95%_	**0.0002**	**0.013**	0.925
	V_110%_	0.126	0.150	0.274
CTV_IMC	V_95%_	**0.003**	**0.004**	0.400
	V_110%_	0.067	0.052	0.801
Heart	D_mean_	0.363	0.284	0.955
Ipsi_lung	D_mean_	0.052	0.148	0.972
	D_10%_	0.193	0.383	0.996
	D_20%_	0.678	0.640	0.839
	D_80%_	**0.011**	**0.034**	0.284
Contra_lung	D_mean_	0.053	0.094	0.240

## Data Availability

The datasets used and/or analyzed during the current study are available from the corresponding author on reasonable request.
